# Dream Big: Effects of Capitals, Socioeconomic Status, Negative Culture, and Educational Aspirations Among the Senior High School Student Athletes

**DOI:** 10.3389/fpsyg.2021.601775

**Published:** 2021-03-15

**Authors:** Chia-Wen Lee, Ming-Chia Yeh, Huang-Chia Hung

**Affiliations:** ^1^College of Modern Management, Yango University, Fuzhou, China; ^2^Graduate Institute of Physical Education, National Taiwan Sport University, Taoyuan City, Taiwan; ^3^Department of Physical Education, National Taitung University, Taitung, Taiwan

**Keywords:** educational aspiration, negative culture, capitals, outstanding student athletes, monthly family income

## Abstract

To understand the impact of social, financial, cultural capitals, negative culture, and socioeconomic status of families on educational aspiration in the senior high school student athletes, it will be beneficial to promote their career developments. The purpose of this study is to explore the influence of ethnicity, year of sport experience family income, the educational expectations of significant others, and the three aforementioned types of capital on educational aspiration among the senior high school student athletes. This study was conducted with a sample of 262 U-18 male baseball student athletes. Of the participants, 20.20% had attained the qualifications to play on the national team. The results showed that monthly family income positively affected social capital and positively indirectly affected educational aspirations through social capital, whereas monthly family income negatively affected negative culture and positively affected educational aspirations through negative culture. Moreover, social capital positively affected educational aspirations compared with negative culture negatively affected educational aspirations. The results serve as a reference for the formulation of educational policy as it relates to student athletes.

## Introduction

Academic performance plays a role in adolescent students’ educational attainment and school dropout (Widlund et al., 2018). Senior high school student athletes (student athletes) are pressured to maintain both academic and athletic excellence. In particular, the athletes’ significant others, who are an object of personal identification and imitation such as parents, teachers, or friends, hold high expectations for their academic performance, perceiving good grades to improve one’s socioeconomic prospects and opportunities for further education (Huang, 2011). However, the senior high school athletes often prioritize their sports to the detriment of their studies. This is unsurprising; athletic training is rigorous, and poor performance on the field harms both their athletic career and prospects for further education (Hung, 2016). Furthermore, some senior high school athletes, who are concerned with their living expenses and the cost of tuition should they enroll in a university, opt for direct employment instead. By contrast, other senior high school athletes continue their studies with the aspiration of obtaining tertiary qualifications and advancing their careers (Göllner et al., 2018). A longitudinal study corroborated the popular belief that academic excellence increases one’s professional and socioeconomic prospects (Marsh and Kleitman, 2005; Hung and Chen, 2010; Göllner et al., 2018).

Regarding their educational aspiration, Coleman (1988) noted that such aspiration is affected by an individual’s social, cultural, and financial capital. Previous studies have demonstrated that consistent social capital – from parents, teachers, and peers – drives student athletes to persist in their training (Fawcett et al., 2009; Hung, 2012; Zheng and Hung, 2018; Hasan et al., 2019). Social capital manifests as parents investing resources – including time, money, and energy – to support their children’s educational goals (Xie, 2014). Similarly, social capital is used when student athletes acquire resources through their relationships with others, whether in their studies, careers, or athletic training (Carron et al., 1996; Li, 2007; Fawcett et al., 2009; Hung, 2016). For instance, educators facilitate the establishment of students’ professional career (Jensen and Jetten, 2015). Student athletes can also acquire cultural capital from being socialized into the tastes and practices of those in a higher socioeconomic bracket, thus allowing them to better utilize their educational achievements (Li and Yu, 2005). Finally, financial capital is crucial for student athletes because playing a sport is expensive. With ample financial resources, student athletes can participate more in their sport, thereby allowing them to acquire more experience, hone their athletic skills, and advance their athletic careers (Jagsi et al., 2009). Therefore, a student athlete’s degree of social, cultural, and financial capital affects their educational aspirations.

When selecting a university to attend, students consider factors such as whether they can continue to compete and train in their sport, their family circumstances, the cost of tuition, and their living expenses (Li and Yu, 2005; Marsh and Kleitman, 2005; Jagsi et al., 2009; Hung and Chen, 2010; Göllner et al., 2018). A student athlete’s demographic characteristics, level of aspiration, and access to capital determine their likelihood of terminating their athletic career (Hung, 2016). However, student athletes in Taiwan represent different socioeconomic backgrounds and may have different influence of social, cultural, financial capital, and negative culture on educational aspiration (Zhang, 2006, 2015; Hwang and Yang, 2007; Su and Hwang, 2009; Hung, 2012, 2016; Hung and Lee, 2013). Therefore, the purpose of this study is to investigate effects of ethnicity, year of sport experiences, family income, negative culture, and the three aforementioned types of capitals on educational aspiration among the senior high school student athletes.

*Hypothesis* 1. Student athletes with better social capital will have higher educational aspiration.
*Hypothesis* 2. Student athletes with better financial capital will have higher educational aspiration.
*Hypothesis* 3. Student athletes with better culture capital will have higher educational aspiration.
*Hypothesis* 4. Student athletes with lower negative culture participation will have higher educational aspiration.

Our aim mainly contributed to the growing literature by conducting the research for capitals, negative culture, and socioeconomic status of families on educational aspiration. It is the first to combine these variables in the context of educational aspiration among senior high school students. The study also provides a new perspective that the socioeconomic differences of ethnic groups have no influences on students’ educational aspirations among the student athletes. Practically, the coaches, teachers, parents, and school faculties can take our findings to be solid evidence to make effective strategies to promote educational aspiration among the senior high student athletes.

## Mateials and Methods

### Participants

The study first passed the IRB examination, acquire the consent of parents, and participants were obtained before the questionnaire. The participants were (1) baseball players from a sports school that was affiliated with the National Taitung University and (2) the senior high school students participating in the 2020 East Coast Baseball League U-18 invitational tournament. A total of 282 questionnaires were distributed, excluding the invalid questionnaires that were missed. We obtained 262 valid questionnaires, average age was 17.34 ± 0.89, and 20.20% of the participants had attained the qualifications to play on the national team.

### Measurements

#### Background

The questionnaire inquired into the following variables.

1. Ethnic group: either Han Chinese or indigenous Taiwanese. Indigenous ethnicity was used as the control in regression analysis.
2. Years of playing the sport.
3. Monthly family income: scored from 1 to 5 for <NT$20,000, NT$20,001–NT$40,000, NT$40,001–NT$60,000, NT$60,001–NT$80,000, and >NT$80001, respectively.

#### Capitals and Negative Culture

We consulted relevant literature to measure the three types of capital (Wu, 1999; Li and Yu, 2005; Zhang, 2006, 2015; Su and Hwang, 2009; Hasan et al., 2019). Social capital was measured by asking student athletes to rate, on a five-point Likert-type scale from strongly disagree (1) to strongly agree (5), how true the following three statements were: “My family cares about my studies,” “My family discusses school affairs with me,” and “My family discusses my plans for the future with me.” Cultural capital was measured using ratings for the following four statements: “I go to bookstores,” “I listen to classical music,” “I attend concerts or plays,” “I visit museums, in particular, art museums,” and “I visit various exhibitions, such as art exhibitions and book exhibitions.” Financial capital was measured using the statements “I own athletic clothing,” “I own sporting equipment,” and “I own sporting accessories.” We also asked about cultural influences that impede socioeconomic advancement (negative cultural factors) by using the statements “I play video games regularly,” “I smoke regularly,” and “I have a rich nightlife” (Cronbach’s α = 0.71).

#### Educational Aspiration

A questionnaire of educational aspiration was assessed which degree student athletes want to have a high school, a university, a master, or a doctor degree after graduating from the senior high school (Hwang and Yang, 2007). We switched the educational aspiration into the educational year, such as 12 for high school, 16 for university, 18 for master, and 21 for doctor degree.

### Data Analysis

The distributions of demographic variables were analyzed using descriptive statistics. A regression analysis was used to analyze the effect of social capital, cultural capital, financial capital, and negative cultural factors on educational aspiration after controlling for ethnicity, years of playing the sport, and monthly family income. The regression analysis also is used to verify the relevant theoretical hypotheses involved in the causal model and to explore the causal mechanism of the differences in educational aspiration from different variables, namely, path analysis. In this path analysis, the standardized regression coefficient (β) is the path coefficient (Lin, 1976).

## Results

### Distribution of Background Variables

Among the student athletes, 36.70% were Han Chinese, (63.30% were aboriginal Taiwanese), and the average number of years of playing the sport was 7.46 ± 1.92 years. Parents, coaches, and peers expected respondents to pursue their education for, on average, 15.97 ± 1.00, 15.97 ± 1.04, and 15.92 ± 1.28 years, respectively.

### Influence on Educational Aspiration

In Table 1, when social capital was used as an intermediary variable in a new regression model (1), social capital was significantly and positively affected by the background variable of monthly family income (β = 0.19, *t* = 2.84, *p* < 0.05); 3.20% of the variation in educational aspiration was explained in this model.

**TABLE 1:** The regression analysis of educational aspiration in senior high student athletes.

	Social Capital (1)						Negative Culture (2)						Educational Aspiration (3)						Educational Aspiration (4)					
				
Variables	*B*	*SE*	β	*t*	95%CI		*B*	*SE*	β	*t*	95%CI		*B*	*SE*	β	*t*	95%CI		*B*	*SE*	β	*t*	95%CI	
							
					L	H					L	H					L	H					L	H
Han (Indigenous as reference)	−0.14	0.10	−0.10	−1.40	−0.34	0.06	0.04	0.10	0.03	0.42	−0.15	0.24	0.29	0.18	0.10	1.51	−0.09	0.64	0.35	0.18	0.13	1.51	0.00	0.71
Years of sport participation	−0.01	0.03	−0.02	−0.36	−0.05	0.04	−0.03	0.02	−0.07	−1.11	−0.07	0.02	0.07	0.04	0.10	1.52	−0.02	0.15	0.06	0.04	0.08	1.53	−0.03	0.14
Monthly family income	0.12	0.04	**0.19**	2.84*	0.04	0.20	−0.10	0.04	−**0.15**	−2.18*	−0.17	−0.01	0.14	0.08	0.12	1.79	−0.01	0.29	0.06	0.08	0.05	1.80	−0.10	0.21
Social capital																			0.32	0.12	**0.17**	2.59*	0.08	0.55
Cultural capital																			0.08	0.09	0.06	0.97	−0.09	0.25
Financial capital																			−0.02	0.10	−0.01	−0.18	−0.21	0.17
Negative culture																			−0.33	0.11	−**0.18**	−2.94*	−0.56	−0.10
*R Square*	0.032						0.025						0.041						0.114					

*p < 0.05. Those bold values meant *p* < 0.05.

Second, when negative cultural factors were used as an intermediary variable in a new regression model (2), educational aspiration was significantly and negatively affected by the background variable of monthly family income (β = −0.15, *t* = 2.18, *p* < 0.05); 2.50% of the variation in educational aspiration was explained in this model.

Third, the regression analysis model of educational aspiration (3) was not significantly affected by the background variables of ethnicity, years of playing the sport, or monthly family income; they explained only 4.10% of the variation in educational aspiration.

Finally, after social capital, cultural capital, financial capital, and negative cultural factors were included in the regression model (4), educational aspiration was significantly and positively affected by social capital (β = 0.17, *t* = 2.59, *p* < 0.05) but significantly and negatively affected by negative cultural factors (β = −0.18, *t* = −2.94, *p* < 0.05); 11.40% of the variation in educational aspiration was explained in this model.

Overall, the results of regression model revealed that student athletes with better social capital and lower negative culture participation have higher educational aspiration, so Hypotheses 1 and 4 were supported and Hypotheses 2 and 3 were not supported.

### A Path Analysis of Educational Aspirations

**FIGURE 1:**
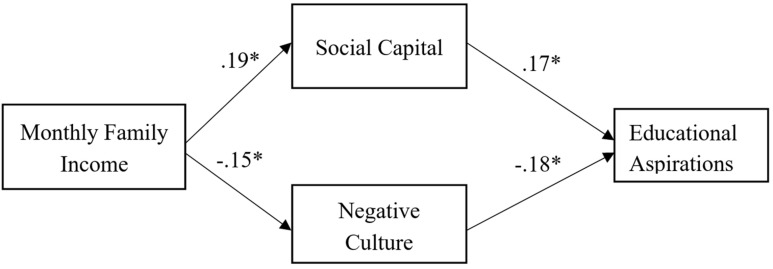
A path analysis of educational aspiration.

In Figure 1, the results showed that monthly family income positively affected social capital (β = 0.19, *p* < 0.05) and positively indirectly affected educational aspirations through social capital (β = 0.03, *p* < 0.05), whereas monthly family income negatively affected negative culture (β = −0.15, *p* < 0.05) and positively affected educational aspirations (β = 0.03, *p* < 0.05) through negative culture. Moreover, social capital positively affected educational aspirations (β = 0.17, *p* < 0.05) compared with negative culture negatively affected educational aspirations (β = −0.18, *p* < 0.05). The model fit indices of the path analysis were 11.40% of the variation in educational aspiration (*R*^2^ = 0.114, *F* = 4.53, *p* < 0.05).

## Discussion

Our results indicate that for the senior high school athletes, a higher monthly family income indirectly increases educational aspiration by increasing social capital and decreasing the influence of negative cultural factors. This result is consistent with the findings in the literature (Sewell et al., 1970; Naidoo et al., 1998; Lin, 2016; Zheng and Hung, 2018) that social capital facilitates career development and the achievement of educational goals. We measured social capital with respect to student athletes’ academic performance, school life, and career development by using the questions of “My family cares about my studies,” “My family discusses school affairs with me,” and “My family discusses my plans for the future with me,” respectively. Parents from higher-income families tend to devote substantial time and effort to their children’s education, particularly by discussing their children’s educational goals with them (Coleman, 1988; Zhou, 2006; Zhang, 2015). Our results further confirm the importance of parental support and interaction on their children’s education.

Student athletes from lower-income families had greater contact with negative cultural factors, which reduced their educational aspiration. We measured the degree of negative cultural factors by asking respondents how much they smoked, were addicted to video games, and indulged in nightlife; these activities distract student athletes from learning and training. This result is consistent with those of previous studies that exposure to high levels of cultural factors makes students more likely to leave a bad impression on teachers, interact poorly with their parents, perform poorly in school, and be less ambitious (Li and Huang, 2004; Huang and Chen, 2005). Negative cultural factors are thus important and especially common in student athletes with a low socioeconomic status. Because parents with a low socioeconomic status have limited resources and knowledge in guiding their children’s learning and career development, schools and social welfare units should intervene to provide such guidance (Lin and Huang, 2008).

All of the regression models were low which is explained in the 2.5–11% in variation These results are consistent with previous studies (Chen and Cheng, 2000; Lee and Hwang, 2004). Lee and Hwang (2004) suggested that to add more background variables or mediating variables could increase the explanatory power of the model. Although this study conducted three background variable of ethnic groups, year of sport participant, and monthly family income, the explanatory power of the model is lower. We speculated that there may be other more important changes that affect students’ educational aspirations, which needs to be further studied.

## Conclusion and Suggestions

Student participation in sports is marked by inequality due to inequalities in society (Lin, 2016). A student athlete’s socioeconomic prospects are limited by the socioeconomic status of their family, which cannot be completely overcome by the athlete’s talent and hard work (Guillet et al., 2002). Moreover, student athletes from low socioeconomic status tend to have different views on gender roles, the family, and work due to a cultural milieu that differs from that of the general population (Harrison and Lawrence, 2003). Their career choice is affected by their family situation (Naidoo et al., 1998). Therefore, their educational aspiration is affected by hard work and talent at the personal level, the social capital from parent–child interaction, and exposure to negative cultural factors. Generally, socioeconomic status, as reflected in monthly family income, directly affects the social capital that athletes have and the negative cultural factors that they are exposed.

Educational aspiration in the senior high school student athletes increases with higher family monthly income, greater social capital, and less exposure to negative cultural factors. Educational aspiration can be improved in student athletes through guidance from teachers, interactions with parents, and less exposure to negative cultural factors. School faculties should establish a platform for parents to interact with their children and other parents and cultivate appropriate everyday habits in student athletes and encourage them to engage in meaningful activities during their spare time, such as participating in club activities (Li and Huang, 2004). To participate in leisure activities can also increase student athletes’ feeling of accomplishment and personal development (Kelly and Godbey, 1992). School faculties and parents should give more guidance to enhance student athletes’ desire to pursue their educational aspirations.

A long-term study of the Wisconsin model demonstrated that family socioeconomic status affects individual achievement and income (Sewell et al., 2004). Educational aspiration is a key to the future success of student athletes. In this study of student athletes, not only social capital but also negative cultural factors were key determinants of educational aspiration after background factors such as ethnicity and family income were controlled and the three types of capital were included as intermediary variables. Negative cultural factors stem from peer influences, and the influence of peers has been suggested by findings that students perform better in school if members of their peer group expect each other to succeed academically, and classmates with the same educational expectations tend to cluster (Raabe and Wölfer, 2019). Therefore, for a more comprehensive understanding of educational aspiration in student athletes, future studies can analyze more determinants, such as family socioeconomic status and the influence of peers and coaches.

The study should be interpreted along with its limitations. The sample of the study may be representative of economic advantage male student athlete in Taiwan since professional baseball is played by males only; however, the finding may limit external validity for other settings or among any subset of our sample. Resources of each professional sport is unique – thus the process of emergence of social capital in each context. We recommend further research to concern different professional student athletes for explanation of educational aspiration.

## Data Availability Statement

The raw data supporting the conclusions of this article will be made available by the authors, without undue reservation.

## Ethics Statement

The studies involving human participants were reviewed and approved by the National Cheng Kung University Human Research Ethics Committee. Written informed consent to participate in this study was provided by the participants’ legal guardian/next of kin.

## Author Contributions

H-CH and C-WL conceptualized the study, developed the methodology, analyzed the data, and reviewed, edited, and wrote the final manuscript. M-CY, C-WL, and H-CH prepared and wrote the original draft. All authors contributed to the article and approved the submitted version.

## Conflict of Interest

The authors declare that the research was conducted in the absence of any commercial or financial relationships that could be construed as a potential conflict of interest.
